# onto2problog: A Probabilistic Ontology-Mediated Querying System using Probabilistic Logic Programming

**DOI:** 10.1007/s13218-020-00670-x

**Published:** 2020-06-06

**Authors:** Timothy van Bremen, Anton Dries, Jean Christoph Jung

**Affiliations:** 1 KU Leuven, Leuven, Belgium; 2 Universität Bremen, Bremen, Germany

## Abstract

We present onto2problog, a tool that supports ontology-mediated querying of probabilistic data via probabilistic logic programming engines. Our tool supports conjunctive queries on probabilistic data under ontologies encoded in the description logic $\mathcal{ELH}^{dr}$, thus capturing a large part of the OWL 2 EL profile.

## Introduction

The amount of data collected has grown considerably in recent years, but with this so has the uncertainty in this data. For example, sophisticated NLP systems like the Never-Ending Language Learner (NELL) [15] are capable of searching the Internet continuously, extracting information from text into a computer-readable logical form. Yet systems like this are not perfectly accurate—indeed, NELL assigns a score to each extracted fact representing the system’s confidence in its truth. These scores can be viewed as degrees of belief in the truth of these facts: in other words, probabilities in the Bayesian sense. Typically, these probabilistic facts are assumed to be mutually independent, resulting in a *(tuple-independent) probabilistic database* [19].

However, in many cases we have some supplementary domain knowledge in the form of an ontology, which can be considered in conjunction with the probabilistic facts. Motivated by this, Jung and Lutz introduced the framework of *ontology-mediated querying of probabilistic data* (OMQPD): given a set of independent probabilistic facts, an ontology, and a query, evaluate the query on the facts taking into account the supplementary knowledge from the ontology [12]. It is important to note that in this line of work the closed-world assumption that is usually adopted in databases is replaced by the *open-world assumption*, that is, the ontology might imply facts that are not explicitly stated in the initial set provided.

For example, suppose we have two probabilistic facts:

$$\begin{aligned} 0.9&\mapsto \mathrm {DepartmentHead}(alice) \\ 0.4&\mapsto \mathrm {mentors}(alice, charlie) \end{aligned}$$

This expresses the knowledge that Alice is a department head with probability 0.9, and, independently, Alice is a mentor of Charlie with probability 0.4. It gives rise to a distribution on four deterministic databases (Table 1): one in which neither fact is true (with probability $(1-0.9)(1-0.4) = 0.06$), one where both facts are true ($(0.9)(0.4) = 0.36$), and two when exactly one is true ($(0.9)(1-0.4) = 0.54$ and $(1-0.9)(0.4) = 0.04$).

**Table 1:** Different interpretations of the probabilistic facts, their probabilities, and facts induced from the ontology in the university example explained in the text

World $\omega$	$P(\omega )$	Induced facts
$$\mathrm {DepHead}(alice),$$$$\mathrm {mentors}(alice, charlie)$$	$$0.9\cdot (0.4) = 0.36$$	$$\mathrm {Professor}(alice), \mathrm {AcadSup}(alice)$$
$\mathrm {DepHead}(alice)$	$0.9\cdot (1-0.4) = 0.54$	$\mathrm {Professor}(alice)$
$\mathrm {mentors}(alice, charlie)$	$(1-0.9)\cdot 0.4 = 0.04$	$\emptyset$
$\emptyset$	$(1-0.9)\cdot (1-0.4) = 0.06$	$\emptyset$

Abbreviations have been used where clear

Now suppose that we also have the following (entirely deterministic) ontology expressed in the description logic $\mathcal{EL}$:

1 $$\begin{aligned} \mathrm{DepartmentHead}&\sqsubseteq \mathrm{Professor} \end{aligned}$$

2 $$\begin{aligned} \mathrm{Professor} \sqcap \exists \mathrm{mentors} . \top&\sqsubseteq \mathrm{AcademicSupervisor} \end{aligned}$$

Intuitively, this ontology expresses that:

1. All department heads are professors
2. A professor who mentors someone is an academic supervisor

Assume we wish to pose the query:

$$\begin{aligned} \Phi = \mathrm{AcademicSupervisor}(alice). \end{aligned}$$

Evaluating the query directly on the set of probabilistic facts earlier returns a probability of zero, as information relating to the class “$\mathrm{AcademicSupervisor}$” does not appear anywhere in the set. But if we evaluate it in combination with the ontology, we get a probability of 0.36, corresponding to the world in which Alice is both a department head and a mentor of Charlie. Thus, the addition of an ontology can change the results of our query, and in particular, reduce the uncertainty. This underpins the idea of OMQPD.

To the best of our knowledge there are so far only preliminary implementations realizing this framework in practice, such as the one proposed by Schoenfisch and Stuckenschmidt [18]. Unfortunately, this system is incomplete in the sense that it only works for certain *safe* combinations of query and ontology, and only for ontologies in *DL-Lite* [2]. On the other hand, Zese et al. [23] presented semantics for DISPONTE knowledge bases and, based on two algorithms (BUNDLE and TRILL), an implementation for inference on these knowledge bases. DISPONTE knowledge bases are slightly different from the framework considered here in the sense that each axiom in the knowledge base—both facts and ontology—is annotated with an independent probability. They use a type-based semantics orthogonal to ours and thus obtain different probabilities for queries. For an overview about other combinations of uncertainty and description logics, we refer the interested reader to (the related work section of) [10].

Here, we propose the tool onto2problog for the task of OMQPD when the ontology is formulated in the description logic $\mathcal{ELH}^{dr}$ and the query is a *conjunctive query*. Conjunctive queries are a common query language and subsume for example the query $\Phi$ above, but can be more complex, such as

$$\begin{aligned} \psi (x) = \exists y . \mathrm{DepartmentHead}(x) \wedge \mathrm{mentors}(y, x) \end{aligned}$$

which asks for all department heads who are mentored by someone.

Further, $\mathcal{ELH}^{dr}$ (which underlies the OWL 2 EL profile [16]) is the extension of $\mathcal{EL}$  [3] with domain and range restrictions as well as role hierarchies. Thus, beyond statements like (1) and (2) above, in $\mathcal{ELH}^{dr}$ we can write statements like

3 $$\begin{aligned} \mathsf {dom}(\mathrm{mentors})&\sqsubseteq \exists \mathrm{hasDegree}.\mathrm{PhD} \end{aligned}$$

4 $$\begin{aligned} \mathsf {ran}(\mathrm{mentors})&\sqsubseteq \mathrm{Student} \end{aligned}$$

5 $$\begin{aligned} \mathrm{mentors}&\sqsubseteq \mathrm{manages} \end{aligned}$$

expressing that:

3. Anyone who mentors has a PhD
4. Anyone who is mentored is a student
5. Someone who mentors a person also manages that person

In contrast to previous work our tool is *complete* in the sense that it can process all combinations of a query and an ontology. The base of our implementation is the adaptation of the *combined approach* to ontology-mediated querying over deterministic data [14] to the probabilistic setting [20]. It therefore reduces OMQPD in $\mathcal{ELH}^{dr}$ to the task of marginal inference in a *probabilistic logic program*, which has an extensive literature surrounding it with many practical techniques available. In principle, this reduction can be used on top of any off-the-shelf probabilistic logic programming engine; we chose ProbLog 2 [8] for our implementation due to its flexibility and widespread use.^1^

In this paper, we first give some background on ontology-mediated querying of probabilistic data, probabilistic data-bases, and probabilistic logic programs. We then describe the implementation of our system and show how it can be used. Finally, we show an evaluation of our system on the Lehigh University Benchmark. For the technical details of our approach, we refer the reader to our earlier conference paper [20].

## Background

In this section, we provide the formal background of ontology-mediated query answering over probabilistic data. We start by reviewing the description logic $\mathcal{ELH}^{dr}$.

### Ontologies in $\mathcal{ELH}^{dr}$

Fix disjoint countably infinite sets of concept and role names $N_C$ and $N_R$, respectively. Then $\mathcal{EL}$-concepts are formed according to the syntax rule

$$\begin{aligned} C {:}{:}{=} \top \ |\ A\ |\ C \sqcap C\ |\ \exists r . C \end{aligned}$$

where $A \in N_C$ and $r \in N_R$. An $\mathcal{ELH}^{dr}$ *-ontology* (hereafter ontology) is a set of *concept inclusions* $C \sqsubseteq D$, *role inclusions* $r \sqsubseteq s$, *domain restrictions* $\mathsf {dom}(r) \sqsubseteq C$, and *range restrictions* $\mathsf {ran}(r) \sqsubseteq C$, where *C* and *D* are $\mathcal{EL}$-concepts and $r,s\in N_R$. An ABox is a finite set of concept assertions *A*(*a*) and role assertions *r*(*a*, *b*) where $A\in N_C$, $r\in N_R$, and *a*, *b* range over a countably infinite set of individual names $N_I$. We denote with $\mathsf {Ind}(\mathcal{A})$ the set of all individual names that occur in $\mathcal{A}$. The semantics of $\mathcal{ELH}^{dr}$ is defined as usual in terms of interpretations $\mathcal{I} = ({\Delta^{\mathcal{I}}}, \cdot ^{\mathcal{I}})$; we elide a full description here and instead refer the reader to Baader et al. [4] for details. We use standard terminology, e.g., $\mathcal{I}$ is a *model* of $\mathcal{T}$ or $\mathcal{A}$ if it satisfies all the concept and role inclusions as well as domain and range restrictions in $\mathcal{T}$, or all the assertions in $\mathcal{A}$, respectively.

### Ontology-Mediated Querying over Probabilistic Data

Let $N_V$ denote a countably infinite set of *variables* disjoint from $N_I$. Then $N_T = N_V \cup N_I$ forms the set of *terms*. A *conjunctive query (CQ)* $\varphi$ is a first-order formula

$$\begin{aligned} \varphi (\mathbf {x}) = \exists \mathbf {y}. \psi (\mathbf {x},\mathbf {y}), \end{aligned}$$

where $\mathbf {x}$ and $\mathbf {y}$ are tuples of variables in $N_V$, and $\phi (\mathbf {x},\mathbf {y})$ is a conjunction of atoms over signature $N_C\cup N_R$ using terms from $N_T$, but only variables from $\mathbf {x}$ and $\mathbf {y}$. We drop the free variables $\mathbf {x}$ of $\varphi (\mathbf {x})$ whenever no confusion can arise. An *ontology-mediated query (OMQ)* is a pair $(\mathcal{T},\varphi )$ of an ontology $\mathcal{T}$ and a CQ $\varphi$. Given an ABox $\mathcal{A}$, and an OMQ $(\mathcal{T},\varphi )$, we say that a tuple $\mathbf {a}$ of individuals from $\mathcal{A}$ is a *certain answer for* $(\mathcal{T},\varphi )$ *over* $\mathcal{A}$ if $(\mathcal{T},\mathcal{A})\models \varphi (\mathbf {a})$, that is, every model $\mathcal{I}$ of $\mathcal{T}$ and $\mathcal{A}$ satisfies $\mathcal{I} \models \varphi (\mathbf {a})$. The set of all certain answers to $(\mathcal{T},\varphi )$ is denoted by $\mathsf {cert}_{\mathcal{A}}(\mathcal{T},\varphi )$.

Following [12], we use *assertion-independent probabilistic ABoxes (ipABoxes)* to model uncertain data. Formally, an ipABox is a pair $(\mathcal{A},p)$ where $\mathcal{A}$ is a classical ABox and $p:\mathcal{A} \rightarrow [0,1]$ assigns a probability to every assertion in $\mathcal{A}$. An ipABox $(\mathcal{A},p)$ induces a distribution $p(\cdot )$ over possible ABoxes $\mathcal{A} '\subseteq \mathcal{A}$, which is defined by taking

6 $$\begin{aligned} p(\mathcal{A} ') = \Pi _{\alpha \in \mathcal{A} '}p(\alpha )\cdot \Pi _{\alpha \in \mathcal{A} \setminus \mathcal{A} '} (1-p(\alpha )), \end{aligned}$$

for every $\mathcal{A} '\subseteq \mathcal{A}$. The *probability of an answer* $\mathbf {a}$ *to an OMQ* $(\mathcal{T},\varphi )$ *over an ipABox* $(\mathcal{A},p)$ is then defined as:

$$\begin{aligned} Pr_{\mathcal{A},p}(\mathcal{T},\varphi ,\mathbf {a})=\sum _{\mathcal{A} '\subseteq \mathcal{A}, \mathbf {a} \in \mathsf {cert}_{\mathcal{A} '}(\mathcal{T},\varphi )}p(\mathcal{A} '). \end{aligned}$$

The prime inference task here is to *compute answer probabilities*, that is, given an ipABox $(\mathcal{A},p)$ and an OMQ $(\mathcal{T},\varphi )$, compute $Pr_{\mathcal{A},p}(\mathcal{T},\varphi ,\mathbf {a})$ for all answer candidates $\mathbf {a}$.

Coming back to the example from the introduction, the set of probabilistic facts corresponds to the ipABox $(\mathcal{A},p)$ where

$$\begin{aligned} \mathcal{A} = \{\mathrm{DepartmentHead}(alice), \mathrm{mentors}(alice, charlie)\} \end{aligned}$$

and

$$\begin{aligned} p(\mathrm{DepartmentHead}(alice))&= 0.9 \\ p(\mathrm{mentors}(alice, charlie))&= 0.4 \end{aligned}$$

If we denote with $\mathcal{T}$ the ontology from the introduction and let $\varphi (x)$ be the query $\mathrm{AcademicSupervisor}(x)$, we have:

$$\begin{aligned} Pr_{\mathcal{A},p}(\mathcal{T},\mathrm{AcademicSupervisor}(x),alice)=.36. \end{aligned}$$

### Probabilistic Logic Programs

We introduce a variant of probabilistic logic programs that is sufficient for our purposes, though some systems support more features. A *probabilistic logic program (PLP)* is a triple $(\mathcal{F},p,\Pi )$ where $\mathcal{F}$ is a set of facts, $p:\mathcal{F} \rightarrow [0,1]$ assigns a probability to every fact, and $\Pi$ is a *stratified logic program* consisting of rules of the form:

$$\begin{aligned} H \leftarrow B_1,\ldots ,B_m, \lnot B_{m+1},\ldots ,\lnot B_n \end{aligned}$$

where *H* and all $B_i$ are relational atoms over terms. The semantics of PLPs $(\mathcal{F},p,\Pi )$ is defined as follows. The pair $(\mathcal{F},p)$ induces a probability distribution $p(\cdot )$ over subsets $\mathcal{F} '\subseteq \mathcal{F}$ just as in Eq.  (6). Moreover, given a set of facts $\mathcal{F}$ and a set of rules $\Pi$, we denote with $\Pi (\mathcal{F})$ the *minimal supported model* of $\mathcal{F} \cup \Pi$, obtained via the iterated fixed point construction of  [1]. The prime inference task for PLPs is *marginal inference*, that is, given a PLP $(\mathcal{F},p,\Pi )$ and a distinguished goal predicate *G*, compute the *probability of all ground facts *$G(\mathbf {a})$ *under* $(\mathcal{F},p,\Pi )$, which is defined as:

$$\begin{aligned} Pr_{\mathcal{F},p,\Pi }(G(\mathbf {a}))=\sum _{\mathcal{F} '\subseteq \mathcal{F}, G(\mathbf {a})\in \Pi (\mathcal{F} ')} p(\mathcal{F} '). \end{aligned}$$

**Fig. 1:**
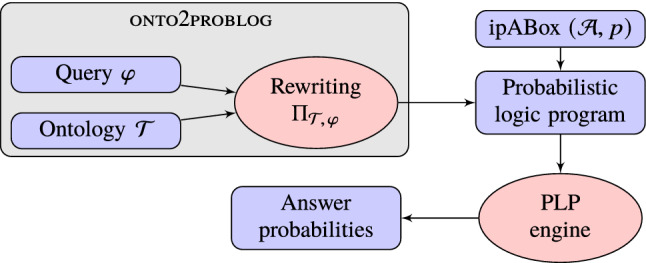
An overview of the the inference pipeline supported by onto2problog

## Our Tool: onto2problog

We have implemented a tool, onto2problog, that enables the use of probabilistic logic programming inference methods for computing answer probabilities of ontology-mediated queries over ipABoxes. The overall architecture of the inference pipeline supported by our tool is depicted in Figure 1. The input of the query answering task consists of the ontology-mediated query (a pair comprising a conjunctive query $\varphi$ and an $\mathcal{ELH}^{dr}$-ontology $\mathcal{T}$), and the probabilistic data given by an ipABox $(\mathcal{A},p)$. Our tool processes only the ontology-mediated query $(\mathcal{T},\varphi )$ and outputs a stratified logic program $\Pi _{\mathcal{T},\varphi }$ with a distinguished goal predicate *G*, which is equivalent to $(\mathcal{T},\varphi )$ in the following sense:

- $(*)$ for every ipABox $(\mathcal{A},p)$ and answer candidate $\mathbf {a}$, we have
  $$\begin{aligned} Pr_{\mathcal{A},p}(\mathcal{T},\varphi ,\mathbf {a})=Pr_{\mathcal{A} ',p,\Pi _{\mathcal{T},\varphi }}(G(\mathbf {a})), \end{aligned}$$
  where $\mathcal{A} '$ is essentially $\mathcal{A}$ in a slightly different representation (described below).

For more concrete information on the structure of $\Pi _{\mathcal{T},\varphi }$, we again refer the reader to our accompanying technical paper [20]. Here, we only stress that its size is polynomial in the sizes of $\mathcal{T}$ and $\varphi$, that the arity of the relation symbols used is bounded by the arity of the query, and that it has only two strata. The use of negation is required to exclude some spurious answers.

We will next give some details on our system and demonstrate its use with the example given earlier in the introduction. We have implemented onto2problog as a Python library, so that it can be called in a flexible and modular way. The ontology is specified in the OWL 2 ontology language (encoded in the standard RDF/XML format [17]), and the query is specified in a simple predicate logic-style syntax.

For example, the fragment of our ontology $\mathcal{T}$ expressing the knowledge that all department heads are professors could be represented as follows in RDF/XML: 
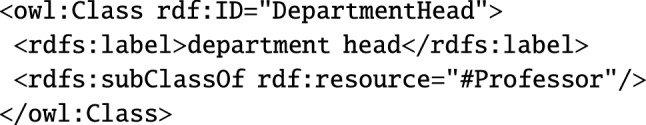


Now suppose we wish to use this ontology and pose the query earlier in the paper asking for all department heads mentored by someone. Then we may specify the query $\psi$ in our Python script in the following way: 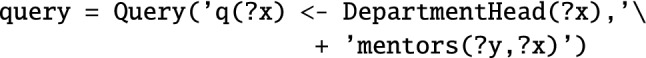 We can then load in the relevant ontology $\mathcal{T}$: 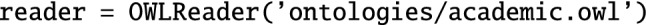 Given $\mathcal{T}$ and $\psi$, onto2problog can then be used to compute the rewriting $\Pi _{\mathcal{T},\psi }$ as described above (after first normalizing the ontology): 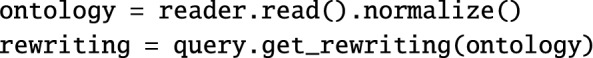 We are now ready to pair the rewriting with an ipABox $(\mathcal{A},p)$. As mentioned above, the rewriting relies on a certain representation of the ABox which we detail next. We represent ipABoxes as strings of probabilistic facts over two fixed predicate names concept and role. For example, the facts $\mathrm{DepartmentHead}(alice)$ and $\mathrm{mentors}(alice,charlie)$ from earlier, along with their probabilities, are specified as the following string: 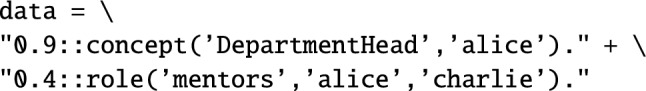 Note that both concept, role, and individual names become constants under this representation. Putting it all together, we get our final probabilistic logic program with the distinguished query predicate q (the name of our query above): 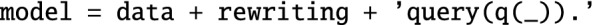 We may now pass this to ProbLog to do the “heavy lifting” of computing the marginal probabilities for the distinguished predicate q in the constructed PLP, producing a list of tuples together with their respective probabilities: 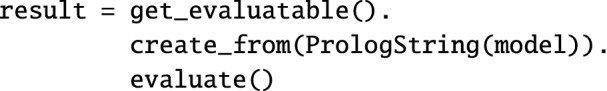 By construction, and in particular because of property $(*)$ above, the results returned are the answers to the original ontology-mediated query task.

ProbLog supports marginal inference via a variety of different algorithms based on knowledge compilation [6], for example, to d-DNNF and SDD. It also supports forward inference in a process known as $T_P$-compilation [22]. Using ProbLog’s Python interface, the user may select which inference method they wish to use in order to evaluate their query.

Our tool together with some documentation and an example is available online at http://www.informatik.uni-bremen.de/~jeanjung/onto2problog.html.

## Evaluation

**Table 2:** Grounding and compilation runtime for the Lehigh University Benchmark queries

	onto2problog				First-order rewriting			
			Classic inference				Classic inference	
Query	Grounding	$T_P$-compilation	Cycle-breaking	Compilation	Grounding	$T_P$-compilation	Cycle-breaking	Compilation
1	0.00	0.00	0.00	0.00	0.04	0.05	0.00	0.00
2	70.14	5.17	0.00	0.00	28.82	0.11	0.00	0.00
3	0.03	0.00	0.00	0.00	0.59	0.67	0.00	0.00
4	25.60	5.73	0.02	0.03	0.88	0.95	0.02	0.03
5	28.24	28.04	1.60	2.53	2.39	5.66	0.40	1.05
6	25.61	71.23	2.92	6.30	4.09	50.12	2.23	5.67
7	78.49	6.26	0.04	0.05	4.53	5.44	0.02	0.05
8	30.24	92.90	3.46	7.47	6.19	71.90	2.54	6.91
9	Timeout	–	–	–	Timeout	–	–	–
10	27.28	4.85	0.00	0.00	4.35	4.63	0.01	0.03
14	0.32	0.12	0.01	0.03	0.20	0.13	0.00	0.00

All times are in seconds. “Timeout” indicates that the procedure took over ten minutes to run

We evaluated onto2problog on a probabilistic version of the Lehigh University Benchmark (LUBM) [9]. LUBM is a benchmark for measuring the performance of semantic knowledge base systems in a consistent manner, comprising an ontology, data generation tool, and a set of test queries. For the purposes of our experiments, we dropped transitive and inverse role declarations from the ontology in order to obtain a valid $\mathcal{ELH}^{dr}$-ontology. Also queries 11, 12, and 13 were deliberately omitted from the test queries as they are specifically designed to test reasoning with inverse and transitive role declarations. We set the parameters of the original data generation tool to generate an ABox of cardinality 15189. Of this, 12260 statements were role assertions and the remainder were concept assertions.

We wrote scripts to transform the assertions generated by the data generation tool to probabilistic facts in ProbLog. As the data from the tool is deterministic by default, we enriched the output by associating each ABox assertion $\alpha$ with an indepedent, uniformly drawn probability $p(\alpha ) \sim \mathcal{U}(0, 1)$ to obtain an ipABox. Finally, using our tool, we computed the rewritings of each of the LUBM queries with respect to the ontology. In the second step we used ProbLog to compute the query probabilities.

We used two different inference methods supported by ProbLog: (1) the “classic” ProbLog inference approach of cycle-breaking and compilation to sentential decision diagrams (SDDs) [21], and (2) $T_{P}$-compilation to SDDs, which avoids the cycle-breaking step altogether through forward inference [22]. Regardless of the method used, ProbLog first computes the *ground* program relevant to the query, that is, it transforms the probabilistic logic program into one using only ground atoms (while returning the same probabilities). We refer to this first phase as the *grounding step*. We refrain from giving more details on the methods (1) and (2) here and instead refer the reader to the aforementioned papers. The runtimes of the computation, divided into the relevant steps, is shown in the left side of Table 2.

We compared onto2problog to an alternative approach to query answering, based on *first-order rewritings*. Informally, first-order rewritings transform the input ontology-mediated query $(\mathcal{T},\varphi )$ into an equivalent first-order query ${{\varphi} _{\mathcal{T}}}$ (or equivalently, a *non-recursive* datalog program). Although first-order rewritings have been used mainly in the classical, that is, non-probabilistic, ontology-mediated query answering, it has been observed that they remain valid also in the probablistic version OMQPD [12]. In the case of the ontology language $\mathcal{EL}$, first-order rewritings are well-studied and it is known that they do not always exist [11]. Thus, they do not provide a complete tool for OMQPD. However, LUBM does not use all features provided by $\mathcal{ELH}^{dr}$. In fact, when dropping the role transitivity axioms, it is essentially formulated in a variant of *DL-Lite*, which implies that for all ontology-mediated queries based on LUBM, first-order rewritings do exist [2]. We therefore manually computed these rewritings and evaluated them using ProbLog as well. The results of this can be found in the right side of Table 2.

**Fig. 2:**
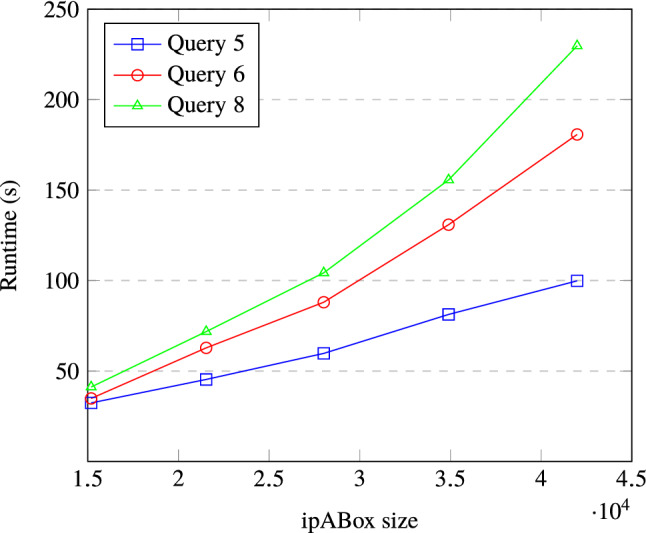
Total inference time on various ipABox sizes, using classic inference

Interestingly, we see that most of the time is spent in the grounding step rather than the knowledge compilation step for each query. These steps correspond to the (deterministic) query answering phase and probability computation phase, respectively. This means that a large amount of time is taken in the computation of the relevant ground program, which is based on SLD-resolution. As SLD-resolution is theoretically not a hard task, we believe this to be the result of inefficiencies in ProbLog’s implementation of grounding which become apparent when dealing with large programs like the ones here.

Moreover, the classic ProbLog inference method of cycle-breaking and compilation to SDDs consistently outperforms $T_P$-compilation. We also observe that first-order rewritings seem to have somewhat better inference times overall, as a trade-off for the incompleteness of this approach. We conclude that in practice, it may be best to first test the first-order rewritability of the query before resorting to the complete approach provided by onto2problog as a second option.

Finally, to get an indication of how our method scales, we examined the total inference time on different ipABox sizes for a subset of the queries in Table 2 for which inference appeared non-trivial. The total inference time here is the sum of grounding, cycle-breaking, and SDD compilation time. The results are shown in Fig. 2. We observe that the runtime increases with ipABox size, but the exact nature of the relationship appears to be dependent on the query in question: the increase is much steeper for query 8 than query 5, for example.

## Conclusion and Future Work

We have presented our tool onto2problog for answering queries over incomplete probablistic data in the presence of ontologies formulated in the description logic $\mathcal{ELH}^{dr}$. The evaluation shows potential for our tool to be used in at least small-scale scenarios. At the same time, it shows that the grounding step can be unexpectedly time-consuming. While it is known that grounding can be expensive in logic programming (see for instance [13] in the context of answer set programming), the PLP $\Pi _{\mathcal{T},\varphi }$ we produce should not be “dangerous” in this sense. We therefore conclude that this is a bottleneck in ProbLog’s implementation, which indeed has been addressed in very recent work [7]. It would be interesting to combine their results with our efforts.

Beyond these improvements to the grounding step, we would like to extend our tool in three directions. First, we want to integrate *first-order rewritings* into our program natively, which on the one hand exhibited better performance in some of our experiments, but on the other hand are incomplete in general. Second, we want to investigate whether our approach can be extended to different ontology languages, such as those in the $\text {Datalog}^{\pm }$ family [5]. Finally, it would be interesting to see whether other capabilities of ProbLog, such as learning, can be transferred to the OMQPD setting.

## Footnotes

1 ProbLog is available for free online at https://dtai.cs.kuleuven.be/problog/.
